# Carbon and Neon Ion Bombardment Induced Smoothing and Surface Relaxation of Titania Nanotubes

**DOI:** 10.3390/nano11092458

**Published:** 2021-09-21

**Authors:** Astrid Kupferer, Michael Mensing, Jan Lehnert, Stephan Mändl, Stefan G. Mayr

**Affiliations:** 1 Leibniz Institute of Surface Engineering (IOM), Permoserstr. 15, 04318 Leipzig, Germany; michael.mensing@isit.fraunhofer.de (M.M.); stephan.maendl@iom-leipzig.de (S.M.); 2 Division of Surface Physics, Felix Bloch Institute for Solid State Physics, Leipzig University, Linnestr. 5, 04103 Leipzig, Germany

**Keywords:** titanium dioxide, nanotubes, diffusion, ion implantation, viscous material flow

## Abstract

Titania nanotube arrays with their enormous surface area are the subject of much attention in diverse fields of research. In the present work, we show that not only 60 keV and 150 keV ion bombardment of amorphous titania nanotube arrays yields defect creation within the tube walls, but it also changes the surface morphology: the surface relaxes and smoothens in accordance with a curvature-driven surface material’s transport mechanism, which is mediated by radiation-induced viscous flow or radiation-enhanced surface diffusion, while the nanotubes act as additional sinks for the particle surface currents. These effects occur independently of the ion species: both carbon and neon ion bombardments result in comparable surface relaxation responses initiated by an ion energy of 60 keV at a fluence of 1 × 10${}^{16}$ ions/cm${}^{2}$. Using atomic force microscopy and contact angle measurements, we thoroughly study the relaxation effects on the surface topography and surface free energy, respectively. Moreover, surface relaxation is accompanied by further amorphization in surface-near regions and a reduction in the mass density, as demonstrated by Raman spectroscopy and X-ray reflectivity. Since ion bombardment can be performed on global and local scales, it constitutes a versatile tool to achieve well-defined and tunable topographies and distinct surface characteristics. Hence, different types of nanotube arrays can be modified for various applications.

## 1. Introduction

Titanium dioxide (TiO${}_{2}$) nanotube arrays constitute a highly adjustable material that is used for various applications. Recent advances in the development of devices for solar energy conversion [1], photocatalysis [2], biomedicine [3] and biosensors [4,5] have been achieved by specifically modifying the starting material: titania nanotube arrays. These arrays comprise general characteristics of bulk TiO${}_{2}$ that are enhanced by the enormous surface area. Moreover, additional unique features emerge from the production process and further post-processing [6]. Most often, electrochemical anodization is the method of choice to produce numerous distinct arrays with a large variation of surface morphology and nanotube lengths. Specific “recipes” are formulated in order to fine-tune surface topographies, surface free energies and mechanical properties [7]. Hence, a manifold of different structures can be realized, ranging from free-standing, double-walled or bamboo-type nanotubes to single nanotubes with large spacings and nanoporous arrays [8]. All of these types of arrays have in common that the self-organized growth introduces an amorphous crystal structure and high stresses. In order to change the crystallinity, high temperature treatments are applied to transform the nanotubes in anatase or rutile phases. In this way, stresses within the nanotube structures are partly relieved due to thermal diffusion of defects [9].

In recent years, several groups have reported on alternative routes beyond annealing to either change crystallinity or reduce material stresses. Recently, Wawrzyniak et al. showed that laser irradiation induces beneficial changes in surface topography and crystallinity [10]. Although an improvement in mechanical stability was reported upon irradiation, the nanotube surface structure was destroyed. However, surface stresses were eased, and the introduced laser energy led to a significant material flow.

Ion implantation has been proven to be a functional tool in order to tailor titania nanotube surface characteristics. In 2005, Ghicov et al. implanted nitrogen ions and observed augmented photocurrents due to the incorporated dopants and band gap shifting [11,12]. Furthermore, surface morphology changed, and a “sponge-like” surface structure was obtained. Surprisingly, Zhou et al. used nitrogen implantation to alter titania nanotubes but did not observed any structural changes [13]. This shows that currently, a concise picture of the consequences of energetic ion irradiation on titania nanotube arrays is not present.

In this study, we investigated the effects of low-energy ion bombardment on the surface structure and morphology of amorphous titania nanotube arrays: carbon and neon ion bombardment with acceleration energies of 60 keV and fluences of 1 × 10${}^{16}$ ions/cm${}^{2}$ induced surface smoothing. Atomic force microscopy revealed that the underlying effect of the structural relaxation is a viscous material flow. Using Raman spectroscopy and X-ray reflectivity, we gained further insight into the modification of the amorphous structure. We detected a nearly complete amorphization of the arrays, and the material density decreased, as implantation introduced point defects that led to swelling behavior. Thus, ion bombardment constitutes a powerful tool that can be used to modify surface morphology and reduce surface stresses globally or locally using masks. Moreover, doping effects can be avoided by choosing noble gases.

## 2. Materials and Methods

### 2.1. Titania Nanotube Array Fabrication

Free standing titania nanotube (NT) arrays were prepared at room temperature using electrochemical anodization, which induced a self-organization process. Therefore, titanium foil (Advent Research Materials Ltd, Oxford, England, 0.1 mm thickness, 99.6+% purity) was cleaned in distilled water and isopropyl alcohol in ultrasonic bath for 10 min each. An organic electrolyte consisting of ethylene glycol (Carl Roth GmbH & Co. KG, Karlsruhe, Germany, for synthesis), 2 vol% distilled water and 0.6 wt% ammonium fluoride (EMSURE^®^ACS, Merck KgaA, Darmstadt, Germany) was used. Titania NT arrays formed on the titanium foil by anodizing at 70 V for 6 min. A platinum mesh, distanced by 45 mm, acted as cathode. To stabilize and cure the titania NTs, the arrays were stored overnight in ethylene glycol. The day after, arrays were cleaned in distilled water in ultrasonic bath for 10 min to remove organic residues.

### 2.2. Ion Bombardment

Ion bombardment of mass separated ${}^{12}$Carbon (C) and ${}^{20}$Neon (Ne) ions was realized using an IMC-200 ion implanter (ion beam services S.A., Peynier, France) with an acceleration voltage of 60 keV or 150 keV yielding sub-surface or deeper implantation profiles, respectively. A standard target tilt angle of 7${}^{{}^{\circ}}$ prevented channeling effects. The energy deposition during ion bombardment was small enough to restrict substrate heating to 50 ${}^{{}^{\circ}}$C or less. Either a low fluence of 8 × 10${}^{14}$ ions/cm${}^{2}$ C or a high fluence of 1 × 10${}^{16}$ ions/cm${}^{2}$ C at 60 keV was applied. Using a combination of both acceleration voltages (60 keV and 150 keV) with a fluence of 1 × 10${}^{16}$ ions/cm${}^{2}$ C each, a more continuous profile of implanted atoms was achieved. For comparative purposes, Ne ions were used only at a fluence of 1 × 10${}^{16}$ ions/cm${}^{2}$ at 60 keV.

To determine the distributions of implanted ions within the material, stopping and range of ions in matter (SRIM) simulations [14] were performed. In order to obtain more realistic simulation conditions, reduced mass densities of 1.09 g/cm${}^{3}$ measured by X-ray reflectivity, as described below, were applied. The average chemical composition of 27.6 at% titanium, 55.2 at% oxygen, 6.9 at% carbon and 10.3 at% fluorine was estimated with energy dispersive X-ray spectroscopy (Bruker Nano GmbH, Berlin, Germany).

### 2.3. Scanning Electron Microscopy

The surface of pristine and ion bombarded NT arrays was imaged using scanning electron microscopy (SEM, Quanta FEG 200, FEI Munich GmbH, Munich, Germany) at a 10 keV acceleration voltage and 5–6 mm working distance with an Everhart–Thornley secondary electron detector.

### 2.4. Atomic Force Microscopy, Autocorrelation Functions and Power Spectral Densities

We measured the surface topographies of NT arrays using a Veeco Instruments Inc. Dimension^®^ Icon™atomic force microscope (AFM, Bruker Nano GmbH, Berlin, Germany) in tapping mode in air equipped with an OTESPA-R3 tip. Two measurement ranges of 2 μm and 50 μm (in *x*-direction) with aspect ratios of 5:1 (1024 × 204 points, M × N) were scanned to obtain micrographs with high resolution and overview images, respectively. SPIP™ software (Image Metrology A/S) was used to determine the root-mean-square (RMS) surface roughness *S*${}_{RMS}$ to calculate correlation functions and to obtain isotropic area power spectral densities (PSD), as described below. The RMS surface roughness is defined as

(1) $$S_{RMS}=\sqrt{\frac{1}{MN}\sum_{k=1}^{M}\sum_{l=1}^{N}{\left[h\left(x_{k},y_{l}\right)\right]}^{2}},$$

where $h(x_{k},y_{l})$ describes the surface height at point $\left(x_{k},y_{l}\right)$ and was chosen in a scale such that <h>=0. To mathematically determine the effects of the ion fluence on smoothing on larger measurement areas of 50 μm, the autocorrelation function

(2) $$C\left(x,y\right)=\sum_{k=1}^{M-x}\sum_{l=1}^{N-y}\left[h\left(k,l\right)\times h\left(k+x,l+y\right)\right],$$

with *k* and *l* as distances in *x*- and *y*-direction, respectively, was applied and plotted in *x*-direction. Using the Fourier transform (FFT)

(3) $$FFT\left(f_{x},f_{y}\right)=\frac{1}{MN}\sum_{i=1}^{M}\sum_{j=1}^{N}h\left(x_{i},y_{j}\right)exp\left[-2i\pi \left(\frac{x_{i}f_{x}}{M}+\frac{y_{j}f_{y}}{N}\right)\right],$$

the PSD was calculated, which can be identified in the steady state solution, as e.g., shown by Mayr and Averback [15], with

(4) $$PSD\left(f\right)=\left(D\left(f\right)\right)/\left(\sum_{i=1}^{4}\left[a_{i}f^{i}\right]\right),$$

where D(f) denotes the strength of the white noise and $a_{i}\geq 0$ denote constants describing distinct surface relaxation processes, as defined by Herring [16]. For instance, the exponent i=1 corresponds to surface diffusion effects, whereas i=4 is associated with a with surface material transport driven by surface curvature [17,18,19].

### 2.5. Raman Spectroscopy

A Horiba LabRam HR Evolution spectrometer (Horiba GmbH) combined with an Olympus MPlan N 100×/0.90 objective was used with a 473 nm laser and an intensity of 0.12 mW on the surface. With a resolution of 0.5 cm${}^{-1}$ (1800 L mm${}^{-1}$ grid), spectra were recorded in the range of 100 cm${}^{-1}$–2000 cm${}^{-1}$. Spectra were background subtracted, and the G peak position and the full-width at half-maximum (FWHM) of the G peak (fitted with double Voigt profile) were determined using a self-written Python script (SciPy Library, “scipy.optimize.curve_fit”) [20,21].

### 2.6. Contact Angle Measurements and Surface Free Energy

We carried out contact angle measurements with one inorganic and two organic fluids to obtain the surface free energy (SFE) according to the Owens–Wendt–Rabel–Kaelble method [22,23]. Water, ethylene glycol and diiodomethane were applied to the surfaces in a contact angle measurement system G2 (A.KRÜSS Optronic GmbH, Hamburg, Germany) combined with the Drop Shape Analyzer DSA 2.5 software.

### 2.7. X-ray Reflectivity

To determine the effect of the applied ion fluences on the density of the material, we performed X-ray reflectivity (XRR) measurements (Cu K$\alpha_{1}$-line, Seifert XRD 3003 PTS, Richard Seifert & Co GmbH & Co KG, Ahrensburg, Germany). The spectra were normalized and fitted with GenX software, version 2.4.10 [24].

## 3. Results

### Ion Bombardment Effects

Ion bombardment was realized by implanting ${}^{12}$C ions with particle energies of either 60 keV or 150 keV or ${}^{20}$Ne ions with energies of 60 keV, as described above. In order to prevent transitions into anatase or rutile high temperature phases, the substrate temperature was kept below 50 ${}^{{}^{\circ}}$C.

**Figure 1:**
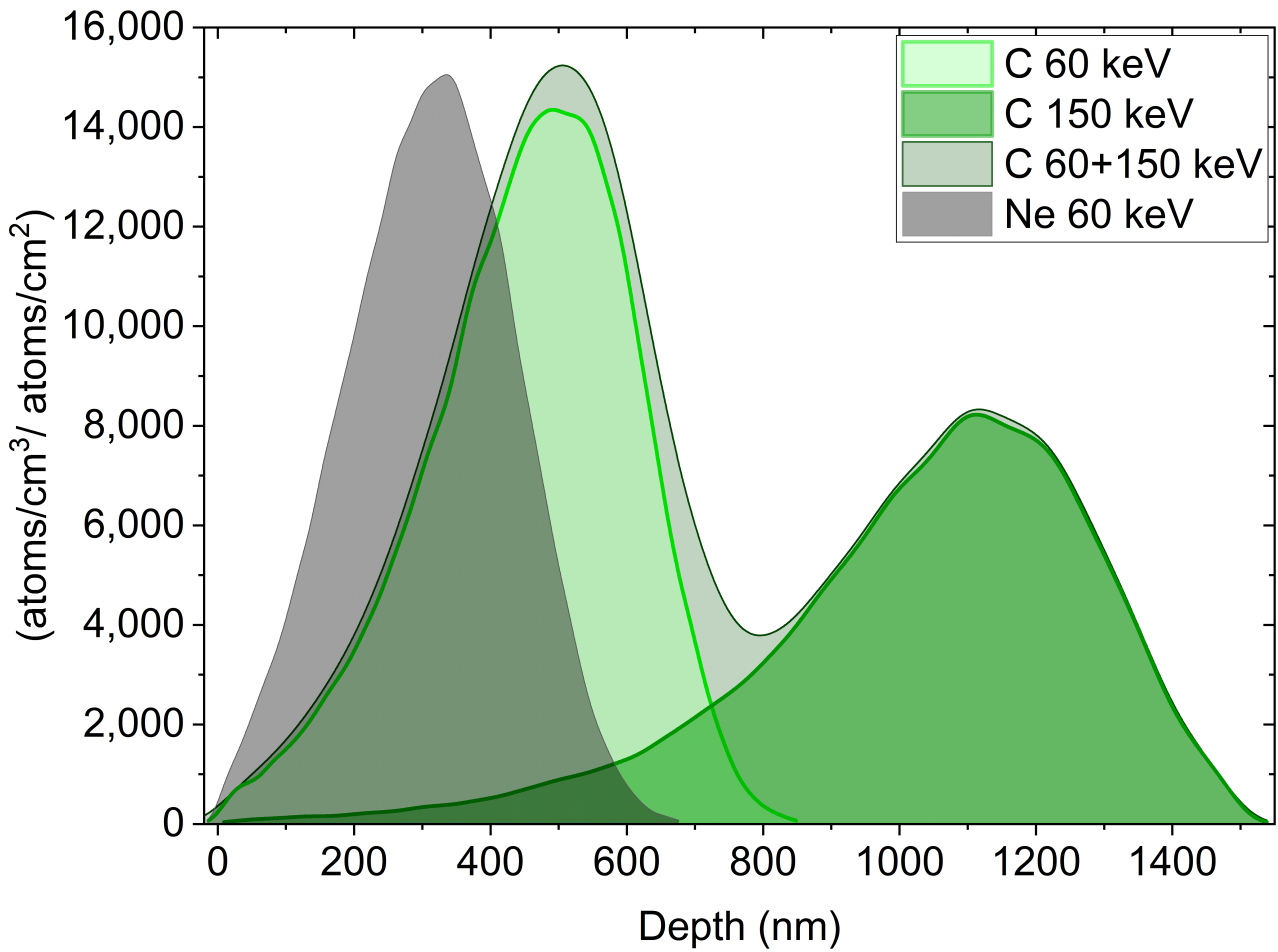
Depth distribution of implanted C and Ne ions in NT arrays obtained with SRIM simulations [14]; the ion beam enters at the left side. The target thickness amounts to 1.5 μm. C ions with 60 keV followed by 150 keV and Ne ions with 60 keV were simulated.

SRIM simulations [14] with effective material densities obtained from XRR illustrate the depth distribution of implanted ions and provide general insight into sputtering damage. In Figure 1, ion ranges of C ions with 60 keV and with 150 keV as well as Ne ions with 60 keV are depicted. The combination of 60 keV followed by 150 keV indeed yields adequate implantation profiles up to 1500 nm in depth. For Ne, the projected ion range is about two-thirds of that for C ions. Interconnected to the ion distribution within the material, defect generation and sputtering can be estimated. The applied low C fluences resulted in negligible sputtering of the arrays; the sputter yield was estimated to be 0.24 atoms/ion for 60 keV ion bombardment and on average 0.18 atoms/ion for combined energies. Therefore, at a maximal fluence of 2 × 10${}^{16}$ ions/cm${}^{2}$ C ions (for combined energies), only about 2–7 atomic layers of the material are sputtered. In contrast, Ne bombardment results in higher sputter yields of 0.65 atoms/ion. Due to the surface-near depth profiles, the number of backscattered ions is higher, and preferential oxygen sputtering of 0.40 atoms/ion is predicted. Hence, Ne ion bombardment induces a higher defect generation in surface-near regions compared to C bombardment.

Since we concentrate on the outermost surface of the NT arrays in this study, these rough sputtering estimates on flat and unstructured surfaces per se present a sufficient basis for assessment of smoothing and relaxation effects upon ion bombardment. Regarding ion implantation effects within NT arrays, a much more complex situation is at play, and enormous inner surface areas have to be considered. We recently provided detailed experimental analysis, analytical models and theoretical frameworks of implantation effects within the NT arrays in several publications [5,25].

**Figure 2:**
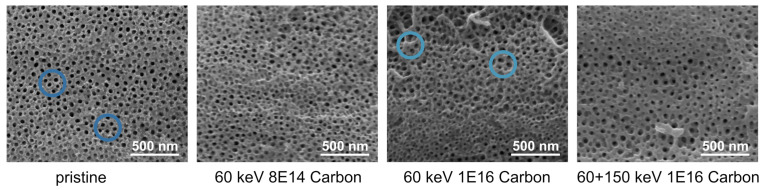
SEM images of NT arrays reveal surface relaxation effects upon ion bombardment with different C ion energies and fluences. Characteristic regions are highlighted with blue circles.

We analyzed the topography of the NT arrays (see Figure 2) before and after ion bombardment by SEM. The NT arrays form perpendicular to the titanium foil underneath and arrange in a hexagonal structure. Without ion bombardment, single NT with clear wall boundaries can be observed; see the highlighted regions in Figure 2. Bombardment with 8 × 10${}^{14}$ ions/cm${}^{2}$ only slightly affects the appearance of the surface. Still, single NT can be detected, and the surface appears rough. In contrast, C ion bombardment with 1 × 10${}^{16}$ ions/cm${}^{2}$ results in the formation of regions with connected NT walls, as highlighted with respective circles in Figure 2. Distinct single NTs are rare. The combination of 60 keV followed by 150 keV with a fluence of 1 × 10${}^{16}$ ions/cm${}^{2}$ C each induces apparent smoothing of the surface. NTs agglomerate and tube walls sinter such that a continuous, more pore-like structure is obtained. The surface appears rather uniform, and defects on the surface (elevated structures stemming from the rolling pattern of the titanium foil underneath) are reduced.

**Figure 3:**
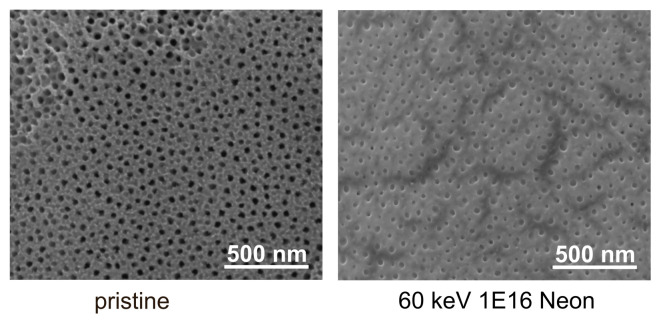
SEM images of NT arrays before and after Ne bombardment with an acceleration energy of 60 keV and a fluence of 1 × 10${}^{16}$ ions/cm${}^{2}$.

Ion bombardment with 1 × 10${}^{16}$ ions/cm${}^{2}$ Ne generates a much more smoothed, pore-like structure, as depicted in Figure 3. The surface appears flattened and homogeneous, since defects are levelled out and all single NT merge.

**Figure 4:**
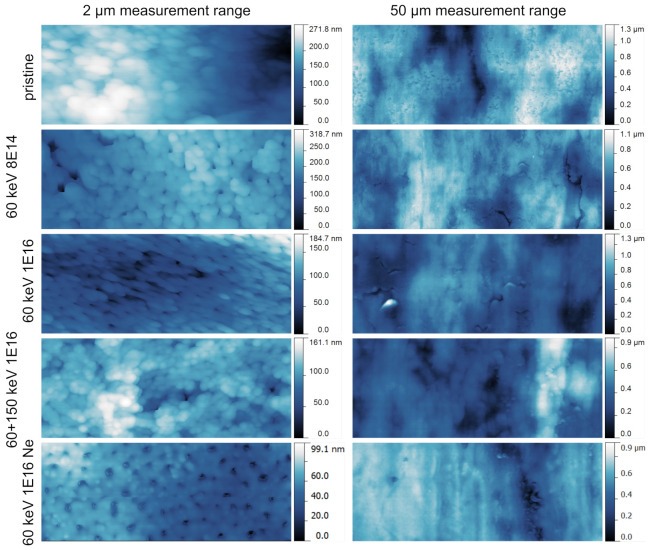
AFM images of NT arrays before and after ion bombardment. In the left column, the topography is measured on a 2 μm range; all images are 1 μm wide. The NT arrays are observed as small, circular cavities (dark blue to black). In the right column, the measurement range was enlarged to 50 μm, and the width of the images amounts to 25 μm. The rolling pattern, originating from the production process of the Ti foil, runs vertical.

We determined the bombardment-induced changes in surface topography using AFM measurements. In the left column in Figure 4, the topography of the NT structure is quantified in a small measurement range of 2 μm. The production process of the Ti foil induces a native rolling pattern. Depending on the position with regard to this pattern, top views (as obtained for a C fluence of 8 × 10${}^{14}$ ions/cm${}^{2}$ and for Ne bombardment) or side views (all other C ion fluences) are acquired. In the top views, the tubular structure clearly emerges, while in side views, the NT openings are partly not visible. Apparently, ion bombardment induces a decrease in the measured height signals. Besides the obvious NT topography, a further substructure on the walls between the pores is observed: small circular elevations are present that diminish with increasing ion fluence. In the right column, overview images with a measurement range of 50 μm are shown. The rolling pattern runs vertically in the images and, in a qualitative view, is only slightly smoothed upon ion bombardment. However, the average root-mean-square (RMS) surface roughness in the 50 μm measurement range decreases with increasing ion fluence. Pristine topographies are characterized by *S*${}_{RMS}$ = 206.7 ± 9.4 nm, whereas NT arrays bombarded with combined energies of C ions exhibit a roughness of *S*${}_{RMS}$ = 143.8 ± 24.8 nm. Hence, the surface roughness of NT arrays is reduced by 30.4%. Moreover, for this large measurement range, the recorded maximal heights of the AFM micrographs decrease, and the surfaces become more homogeneous. Ne bombardment results in an even lower surface roughness of *S*${}_{RMS}$ = 26.0 ± 11.1 nm; hence, a decrease of 87% compared to pristine NT arrays can be observed.

**Figure 5:**
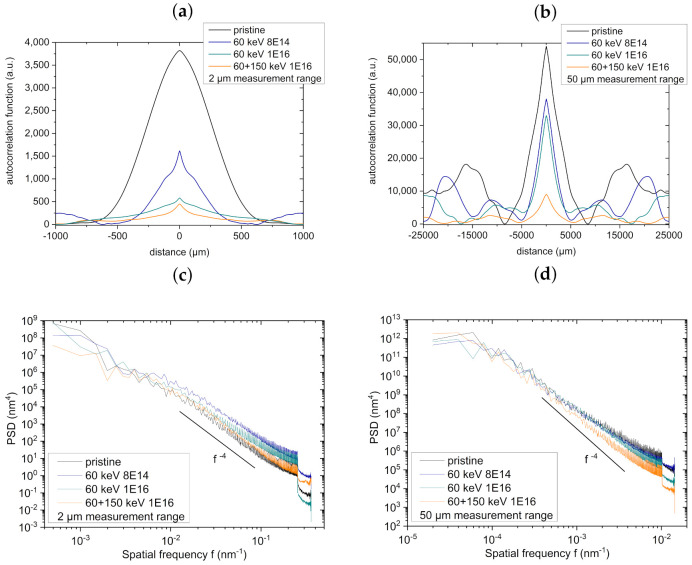
Graphs showing the calculated autocorrelation functions (**a**,**b**) as well as PSDs (**c**,**d**) obtained from AFM images of NT arrays bombarded with C depicted in Figure 4. The surface topography was measured in (**a**,**c**) 2 μm measurement range and (**b**,**d**) 50 μm measurement range. The PSDs obtained from the 2 μm measurement range are nearly identical with prominent *f*${}^{-4}$ behavior, which is in accordance with surface material transport driven by surface curvature.

The autocorrelation functions in Figure 5 quantify the smoothing effect of the C ion bombardment: the total heights of the functions decrease in both ranges. In the 50 μm measurement range, the amplitudes of the topographies decline drastically. Similar conclusions can be drawn from the log–log plot of the isotropic area PSD depending on the spatial frequency. Moreover, the rather small parallel shift in the PSD in the 50 μm measurement range shows the smoothing effect of the C ion bombardment.

The exponents j of the fits obtained in the log–log plot can be associated with Equation (4), in detail $PSD\left(f\right)\propto f^{-j}$. The $f^{-4}$ behavior corresponds to material transport in surface-near regions that is observed for higher frequencies in both measurement ranges. The underlying mechanisms mediating this type of transport may be radiation-induced surface viscous flow or curvature-driven radiation-enhanced surface diffusion, which dominate surface morphology shaping, while nanopores certainly act as sinks for the viscous or diffusive surface flows, respectively [18].

**Figure 6:**
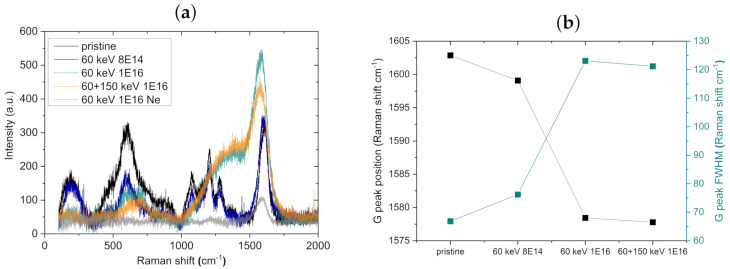
(**a**) Raman spectra of NT arrays before and after C and Ne bombardment. The carbon G peak is located at around 1580 cm${}^{-1}$–1600 cm${}^{-1}$. (**b**) The G peak position and the G peak full-width at half-maximum (FWHM) are plotted. The lines connecting the data points guide the eye. For Ne-bombarded NT arrays, the G peak is located at 1585.6 cm${}^{-1}$. This figure constitutes a compilation of our final set of Raman spectra; data on pristine and 60 keV followed by 150 keV C ion bombardment spectra were already included in Ref. [5].

We carried out Raman measurements to gain insight into the crystalline structure, i.e., the phase composition of the material. In Figure 6a, background-corrected spectra of NT arrays before and after ion bombardment are depicted. Pristine arrays as well as arrays bombarded with 8 × 10${}^{14}$ ions/cm${}^{2}$ at 60 keV show similar peaks in position and widths. At about 612 cm${}^{-1}$ a broad structure is located that is associated with the A${}_{1G}$ mode of the rutile TiO${}_{2}$ polymorph [26,27]. Since the grain size of the crystallites affects the broadness of the bands [28], the width of the 612 cm${}^{-1}$ peak indicates that small rutile nanocrystallites are prominent. Further localized peaks in the range of 100 cm${}^{-1}$–1000 cm${}^{-1}$ are not observable, which is in agreement with a mixture of rutile and amorphous, as already stated by Hardcastle et al. in 2011 [29]. Furthermore, three peaks at about 1080 cm${}^{-1}$, 1200 cm${}^{-1}$ and 1280 cm${}^{-1}$ are present, and these are attributed to the incorporation of organic electrolyte residues due to the applied anodization voltage. For instance, the stretching of C=C may produce the peak at 1080 cm${}^{-1}$ [30], and stretching vibrations of C-F -bonds can result in peaks at 1200 cm${}^{-1}$ [31]. At 1600 cm${}^{-1}$ the carbon G peak is located, and this mainly originates from the incorporated organic electrolyte residues. For higher C fluences, the rutile peak at 612 cm${}^{-1}$ smears out, and an additional shoulder at higher wavelengths appears. This indicates further amorphization. While the peaks near 1080 cm${}^{-1}$, 1200 cm${}^{-1}$ and 1280 cm${}^{-1}$ vanish, a double peak consisting of a broad peak (presumably not the carbon D peak) and the carbon G peak located at around 1355 cm${}^{-1}$ and 1580 cm${}^{-1}$, respectively, appears at an ion energy of 60 keV with a fluence of 1 × 10${}^{16}$ ions/cm${}^{2}$. In Figure 6b, the shift of the G peak position and the broadening of the full-width at half-maximum (FWHM) upon C implantation are illustrated. The G peak shift corresponds to a change in the predominant carbon species: the position at 1600 cm${}^{-1}$ correlates with nanocrystalline graphite [32], occurring presumably due to the organic residues, whereas the peak position at 1580 cm${}^{-1}$ is assigned to graphite [33], originating from compositional patterning produced by implanted C ions [5].

Upon Ne ion bombardment, nearly all peaks vanish. Only a small carbon G peak can be observed at 1585.6 cm${}^{-1}$, which also corresponds to graphite.

One explanation for the continuous decrease in the G peak position as well as the increase in the G peak FWHM is a surface relaxation process upon C ion bombardment. The broadening of the FWHM implies that the nanocrystalline quality of the observed C decreases. Indeed, detailed descriptions of stress-induced effects as well as relaxation based on Raman peak shifts were given by Anastassakis et al. and De Wolf for materials such as silicon and diamond [34,35]. In addition, Nair et al. described changes in Raman spectra of nanostructured CdS thin films activated by ion implantation that underline our findings [36].

Another explanation is connected to our recently reported findings of compositional patterning related to C ion implantation [5]. Upon implantation, a formation and spatial separation of C and O domains arise in the NT walls. The larger C domains might lead to the increase in graphite peaks at 1580 cm${}^{-1}$. However, the impact of the exact penetration of the Raman laser as well as the measurement volume cannot be estimated. Thus, the signal fraction stemming from the C domains is unclear.

**Figure 7:**
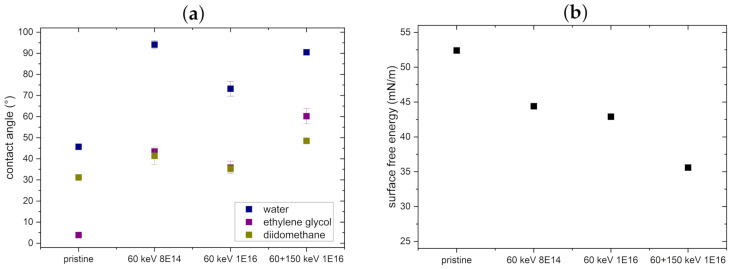
(**a**) Contact angle measurements with water, ethylene glycol and diiodomethane before and after ion bombardment of NT arrays with standard deviation. (**b**) SFEs calculated according to Owens–Wendt–Rabel–Kaelble from the respective contact angles.

In addition, we performed contact angle measurements to obtain the SFE. As depicted in Figure 7, the contact angles increase with increasing ion fluence, while the SFE decreases, again indicating a surface relaxation process [19], as the chemical composition is mainly constant with the implanted C concentration still at the level of doping only.

**Figure 8:**
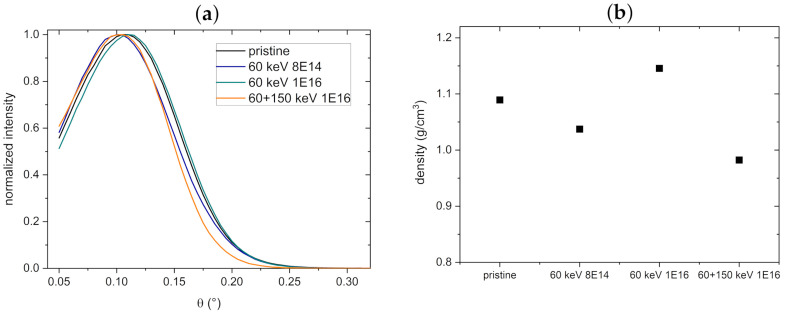
(**a**) Normalized XRR spectra of NT arrays, before and after C bombardment. (**b**) Mass densities obtained from the spectra in (**a**). The standard deviations of the fitted mass densities are lower than 5%.

To determine the effect of ion bombardment on the density of the material, XRR measurements were performed. Spectra and corresponding mass densities are displayed in Figure 8, showing that the mass density for NT arrays decreases by 11% from 1.09 g/cm${}^{3}$ of the pristine NT to 0.98 g/cm${}^{3}$ after bombardment with combined C acceleration energies. Since the substrate height decreases upon implantation [5], we assume that the overall volume of the arrays increases due to the introduced defects, as reported recently [25].

## 4. Discussion

Stresses within the NT arrays result from surface stresses, viz. capillarity due to NT morphology and surface roughness, as well as from the production process of NT arrays: the self-organized growth of NT arrays under applied voltage is expected to generate stresses inside the tubes. To relieve these stresses, different routes can be followed. Annealing at elevated temperatures [37] cures surface stresses but, in general, results in a phase change to anatase or rutile. In our case, the effects of ion bombardment are investigated. Here, only high ion fluences in the order of 1 × 10${}^{17}$ ions/cm${}^{2}$ are expected to notably affect the material’s crystallinity, neglecting a formation of small nanoparticles [38] and super-saturated solutions that are not present here [39]. We concentrate on low-fluence ion bombardment, describing changes in surface morphology and the interactions of accelerated ions with the amorphous titania NT material. Depending on ion species, distinct degrees of surface smoothing and stress relaxation can be obtained.

Panepinto et al. [40] recently described N ion bombardment effects on annealed, anatase, magnetron-sputtered films with lower surface roughness. Apparently, fluences of 1 × 10${}^{16}$ ions/cm${}^{2}$ at 30 keV only marginally affected materials characteristics, such as substrate height and surface morphology. Presumably, magnetron sputtering and subsequent annealing reduced stresses to negligible quantities such that stress relaxation was not observed.

In this study, we modify the distinct NT topography by C and Ne ion bombardment. Smoothing of the surface morphology is demonstrated using SEM and AFM; effects of surface relaxation are confirmed with Raman spectroscopy, SFE estimates and XRR. For both ion species, an energy of 60 keV results in near-surface deposition of implanted ions. In this finite volume, energy is dissipated in collision cascades [41], and defects are created. By means of the resulting enhanced atomic mobility, which can be mediated by radiation-induced viscous flow as well as radiation-enhanced surface diffusion, the system locally relaxes more toward equilibrium, which is manifested by surface smoothing as well as structural relaxation toward crystallinity.

The pronounced surface smoothing of Ne ion bombardment is connected to the higher ion mass and, hence, a more than two times higher collision cross-section compared to C ions [42]. In this way, the volume of maximum defect density is located nearer to the surface with a higher energy deposition at the exact surface position. Consequently, surface relaxation and smoothing effects are enhanced even at low ion fluences. Therefore, bombardment of elements between carbon and neon (nitrogen, oxygen and fluorine) should result in comparable material responses. Surprisingly, neither Ghicov et al. [12] nor Zhou et al. [13] reported such effects, although they used similar NT arrays and the same fluences.

## 5. Conclusions

In conclusion, our results demonstrate that ion bombardment induces not only doping effects in the amorphous titania NT material but mainly surface relaxation and smoothing effects. These effects can be explained on the basis of the transport of curvature-driven surface materials, which is mediated by radiation-induced viscous flow or radiation-enhanced diffusion, while the NTs act as additional sinks for the surface particle currents. Additionally, upon implantation, the titania NT material shows a higher degree of amorphization, correlated with increased defect density and reduced mass density. The species of the ions is irrelevant: for the topography, the bombardment of carbon and neon ions results in similar relaxation effects of NT arrays for comparable fluences. In this way, precise surface engineering of titania NT arrays on either a global or local scale using masks is enabled and paves the way for tailored applications.

## Data Availability

The data presented in this study are available on request from the corresponding author.

## Abbreviations

The following abbreviations are used in this manuscript:

AFM	atomic force microscopy
FFT	fast Fourier transform
FWHM	full-width at half-maximum
NT	nanotube
PSD	power spectral density
RMS	root-mean-square
SEM	scanning electron microscopy
SFE	surface free energy
